# Evaluation of Appropriate Hysteresis Model for Nonlinear Dynamic Analysis of Existing Reinforced Concrete Moment Frames

**DOI:** 10.3390/ma14030524

**Published:** 2021-01-22

**Authors:** Joo-Ki Son, Chang-Hwan Lee

**Affiliations:** Division of Architectural and Fire Protection Engineering, Pukyong National University, 45, Yongso-ro, Nam-gu, Busan 48513, Korea; son961129@naver.com

**Keywords:** nonlinear dynamic analysis, reinforced concrete, moment frame, hysteresis model, energy dissipation

## Abstract

Various seismic analysis methods are being used to predict the response of structures to earthquakes. Although nonlinear dynamic analysis (NDA) is considered an ideal method to represent the most realistic behavior of a structure among these various methods, correct results can be derived only when the analysis model is carefully developed by a knowledgeable person. It is particularly important to properly implement the behavior characteristics depending on the reversed cyclic load in the NDA of a building made of reinforced concrete (RC) moment frames. This study evaluated the hysteresis model suitable for NDA of existing RC moment frames, and 45 analysis models were reviewed, in which the pivot, concrete, and Takeda hysteresis models were applied differently to beams and columns. The pivot model was evaluated as the most reliable hysteresis model for each structural member by comparing and analyzing not only the responses of the entire frame but also the responses of column and beam members focusing on energy dissipation. However, this model can have practical limitations in that the parameters associated with the reinforcement detailing and applied loads need to be defined in detail. The analysis model applying Takeda to the beam, which predicted the average response at a reliable level compared to the reference model, was identified as a practical alternative when it is difficult to apply the pivot model to all frame members.

## 1. Introduction

The Northridge, Kobe, and Kocaeli earthquakes highlighted the limitations of conventional approaches that elastically estimate the response of structures to earthquakes [1]. Accordingly, methods for predicting the response of structures more realistically compared to the conventional elastic approaches have been developed, and nonlinear static analysis (NSA) and nonlinear dynamic analysis (NDA) are widely being used. In particular, the latter can obtain results closest to the actual response [2], which is considered an ideal method when evaluating and designing structures for resistance against earthquakes.

In NDA conducted for existing reinforced concrete (RC) buildings, the behavioral characteristics of members such as pinching, stiffness degradation, and strength degradation depending on the reversed motion can have a significant influence on the response of the structure. In particular, when the seismic-force-resisting system is composed of moment frames, it is more flexible than the shear walls or braced frames, and the behavior of the entire structure is dominated by the hysteresis characteristics of these frame members as it needs to resist seismic forces through the frame action of columns and beams. Therefore, it is important to apply a hysteresis model that can adequately represent the actual behavior of the frame members when performing NDA for the existing RC moment frames.

Various forms of hysteresis models have been developed to date to represent the behavior of RC frame members under reversed cyclic loading. Many of these models have been designed to estimate the frame behavior based on the ductile flexural characteristics [3,4,5,6], and Ozcebe and Saatcioglu proposed an effective model for the estimation of shear behavior [7]. Strojadinovic and Thewalt presented a hysteresis model that can more realistically simulate the changes in energy dissipation compared to existing models, based on strength degradation [8]. In addition, Dowell et al. devised a pivot model that can represent pinching more effectively than the Takeda model, which was commonly used for RC members [9]. Sezen et al. developed a model that considers the effects of the applied axial and shear forces on the column [10], and Zimos et al. designed a hysteresis model suitable for members showing shear behavior after the development of the maximum strength [11]. In contrast, Xu and Zhang presented a shear–flexure interaction model for a column, which adequately represented pinching, stiffness degradation, and strength degradation [12]. Allouzi and Irfanoglu proposed a hysteresis model to adequately describe the dynamic response of RC frames with infill walls, and the predictions performed using this model were close to the experimental results [13]. Furthermore, studies based on the Bouc–Wen model [14,15], which yields a smooth hysteresis curve, have been conducted recently [16,17]. Pelliciari et al. defined the degradation effect by introducing a damage index related to the maximum displacement and dissipated energy, followed by a new model based on the differential equation of the Bouc–Wen model [18]. Likewise, although various hysteresis models have been proposed for RC frame members, studies that have compared and evaluated the developed models are lacking [19].

In this context, we conducted a study to provide useful basic data for selecting an appropriate hysteresis model for NDA of an existing building with an RC moment frame system. To this end, the details of the analysis models prepared for standard schools widely constructed in the 1980s were examined, comprising 45 cases in which pivot, concrete, and Takeda hysteresis models were applied differently to beams and columns. The responses of the entire frame, followed by the responses of the members in the order of columns and beams, were compared and analyzed, focusing on energy dissipation from the NDA results. Subsequently, the hysteresis models suitable for analyzing the RC moment frame were evaluated, and the most reliable or practical model was identified.

## 2. Analysis Models

ETABS [20], a commercial structural analysis program, was used for the analysis, and evaluation was performed using the pivot, concrete, and Takeda models that were determined suitable for RC frame members among the built-in models. The energy factor was set to 0.7 in the concrete model by referring to the characteristics of each hysteresis model presented in the program manual [20]. The parameters for the pivot model were input with reference to Sharma et al. [21].

**Figure 1:**
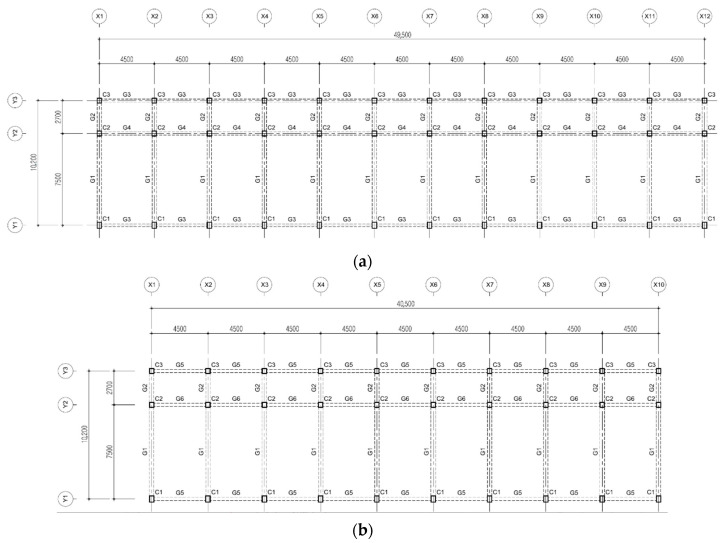
Typical floor plan: (**a**) Types 1 and 3, and (**b**) Type 2.

**Figure 2:**
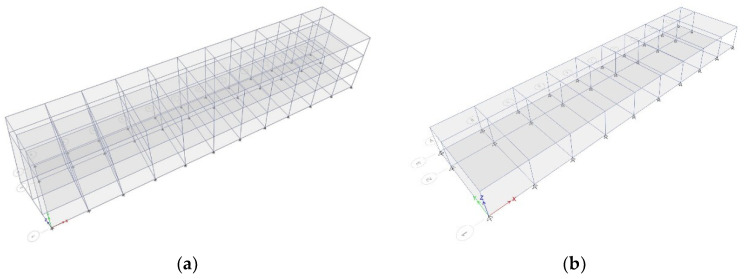

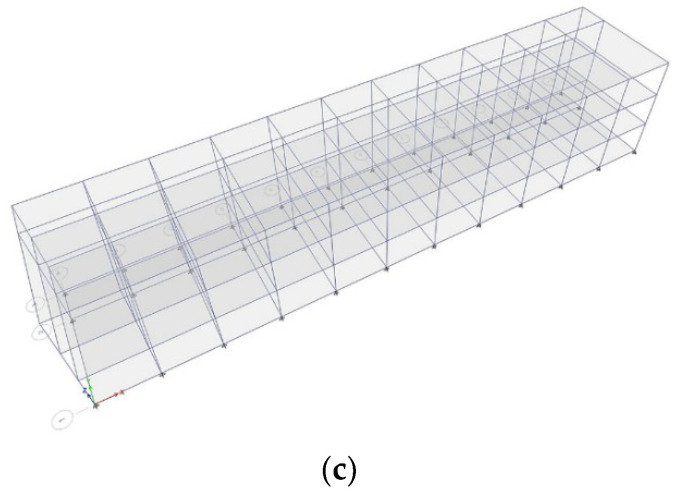
The 3D model of the analysis structure: (**a**) Type 1, (**b**) Type 2, and (**c**) Type 3.

This study was conducted on existing buildings; analysis was performed on standard schools constructed in the 1980s with RC moment frames in Korea. Although there are several types of buildings [22,23,24], the three types presented in Table 1 were selected as the analysis structures by integrating similar types in this study. The height of each story, 3.3 m, was consistent throughout. Although Types 1 and 3 were three-story structures and had the same typical floor plan (Figure 1), there were differences in the load and reinforcement detailing of the sections. Type 2 was a one-story structure, as shown in Figure 2.

**Figure 3:**
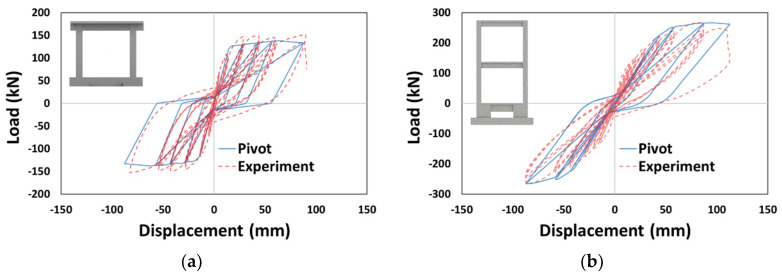
Additional verification for setting a reference model: (**a**) Oh’s frame [26] and (**b**) Lee’s frame [27].

Five analysis models were prepared for each type by applying the hysteresis models to beam and column members differently for the three types of analysis structures, as shown in Table 2. In a preceding study [25] on a two-dimensional frame under quasi-static cyclic loading, the pivot model most closely expressed the experimentally observed behavior. Additional comparative analyses on the single-story Oh’s frame [26] and two-story Lee’s frame [27] in this study showed that the pivot model also predicted the experimental results well, as shown in Figure 3. The P.P., which applied a pivot model involving beams and columns, was set as the reference analysis model in this study based on these findings. Accordingly, the analysis models changed to concrete and Takeda models for columns were named P.C. and P.T., respectively, and those changed to concrete and Takeda models for beams were named C.P. and T.P., respectively.

**Figure 4:**
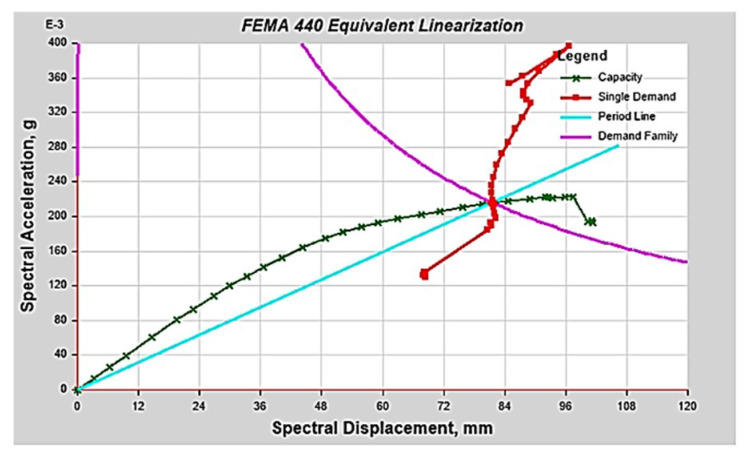
Nonlinear static analysis result (Type 1).

**Figure 5:**
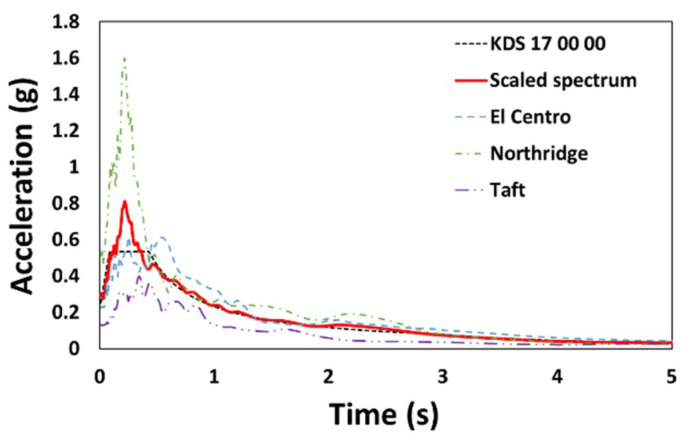
Scaled response spectrum.

As the analysis model was a three-story low-rise building, NDA was performed using three recorded ground motions with dominant acceleration in the short-period region: El Centro, Northridge, and Taft. Prior to the scaling of the selected ground motions, NSA was conducted for each type to pre-evaluate the overall characteristics of the structure. The NSA results for Type 1 considering an earthquake with a 2400-year return period [28] showed that although the performance point was formed, the overstrength and remaining deformation capacity of the structure were limited, as shown in Figure 4. For similar reasons, there were cases in which the analysis was incomplete owing to instability and collapses during NDA for unscaled ground motion. The ground motion was scaled to the level of a 1000-year return period as the purpose of this study was to examine the difference in behavior depending on the applied hysteresis model (Figure 5).

## 3. Results and Discussion

The NSA and NDA results were first compared for each analysis model. The maximum base shear (*V_max_*), maximum displacement (*D_max_*), base shear–displacement curve, energy dissipation, and behavioral characteristics of individual members were compared and analyzed after first evaluating the appropriateness of the analysis results. Five analysis models prepared for each type of structure were analyzed for the three ground motions, resulting in a total of 45 results. The results are described based on Type 1 unless otherwise stated in this section.

### 3.1. Comparison of NSA and NDA Results

The NSA and NDA results for the Type 1 P.P. model are summarized in Table 3. The NDA response to the Taft earthquake was approximately half of that of the NSA, and the base shear and roof displacement were 13.4–20.0% and 7.4–8.2% higher in NDA, respectively, for the two other earthquakes.

### 3.2. Overall Responses

The *V_max_* and *D_max_* for each analysis model are given in Table 4. However, all analysis models showed elastic behavior for the ground motions scaled to the 1000-year return period in Type 2, and the results, considering the ground motion rescaled to the 2400-year return period, are presented accordingly. Compared to P.P., the Type 3 C.P. (El Centro, Y-dir.) showed significant differences with a maximum of 11.5% in *V_max_* and a maximum of 13.0% in *D_max_*. In addition, although Type 1 P.T. (El Centro, X-dir.), Type 2 P.C. (El Centro, Y-dir.), and Type 3 T.P. (El Centro, Y-dir.) showed relatively significant differences from P.P., the differences were relatively small when compared to the difference shown by Type 3 C.P. (El Centro, Y-dir.).

**Figure 6:**
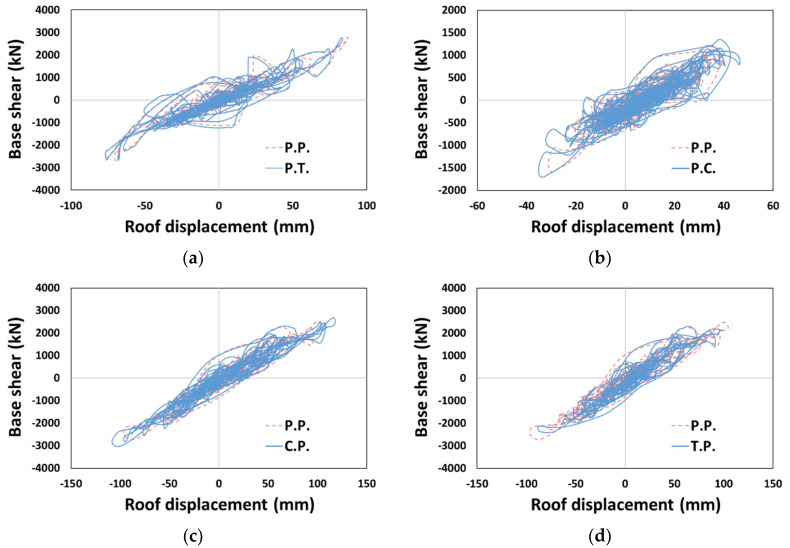
Base shear–displacement curves: (**a**) P.T. (Type 1, El Centro, X-dir.), (**b**) P.C. (Type 2, El Centro, Y-dir.), (**c**) C.P. (Type 3, El Centro, Y-dir.), and (**d**) T.P. (Type 3, El Centro, Y-dir.).

Out of the 45 analysis results, we mainly analyzed the models and types that showed a relatively significant difference from P.P., excluding cases showing elastic behavior. The base shear–roof displacement curves for the representative models of each type are shown in Figure 6, and indicated no evident difference from that of P.P. in the overall response result.

**Figure 7:**
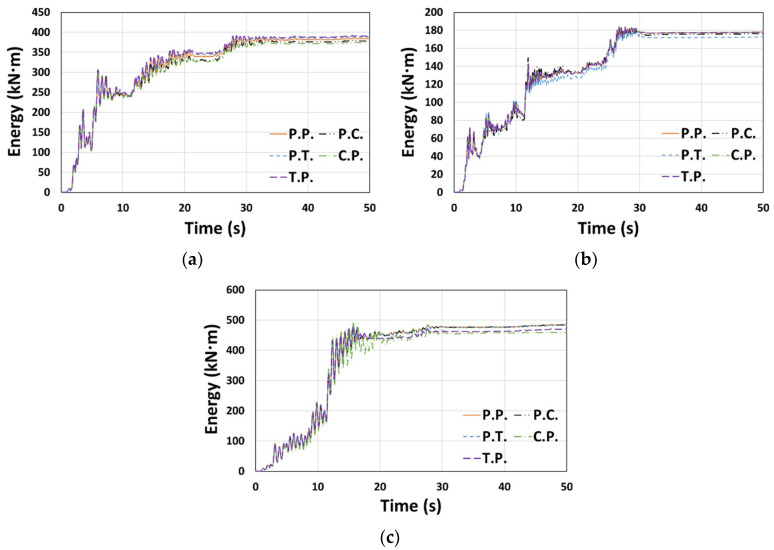
Cumulative dissipated energy: (**a**) Type 1 (El Centro, X-dir.), (**b**) Type 2 (El Centro, Y-dir.), and (**c**) Type 3 (El Centro, Y-dir.).

**Figure 8:**
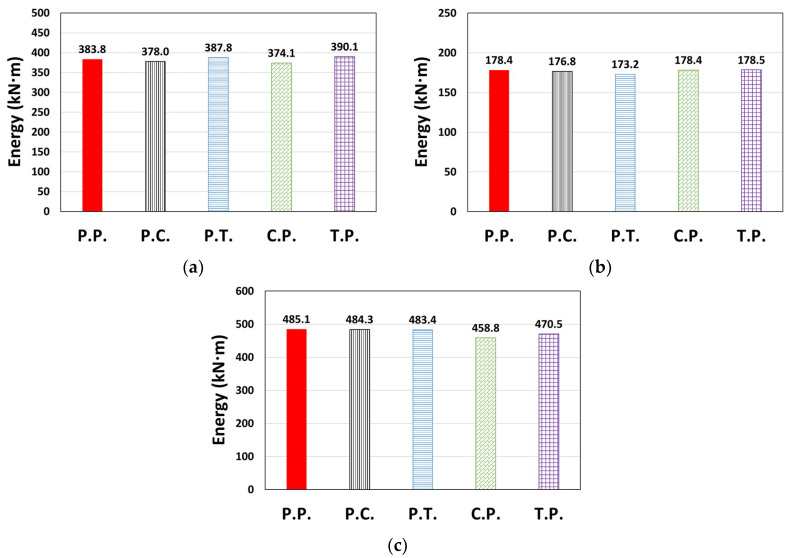
Total dissipated energy (*E_t_*): (**a**) Type 1 (El Centro, X-dir.), (**b**) Type 2 (El Centro, Y-dir.), and (**c**) Type 3 (El Centro, Y-dir.).

The cumulative dissipated energy over time for each type of El Centro ground motion in the X or Y direction is shown in Figure 7, where the models considered in Figure 6 generally show similar results. In terms of the total dissipated energy (*E_t_*) shown in Figure 8, the error compared to the P.P. model was relatively small, ranging from −5.4% to 1.6%.

**Figure 9:**
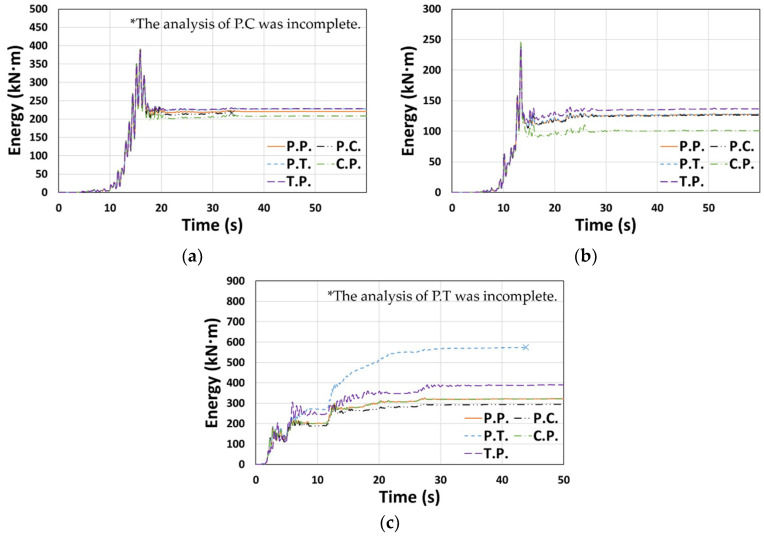
Cumulative dissipated energy: (**a**) Type 3 (Northridge, X-dir.), (**b**) Type 3 (Northridge, Y-dir.), and (**c**) Type 2 (El Centro, X-dir.).

**Figure 10:**
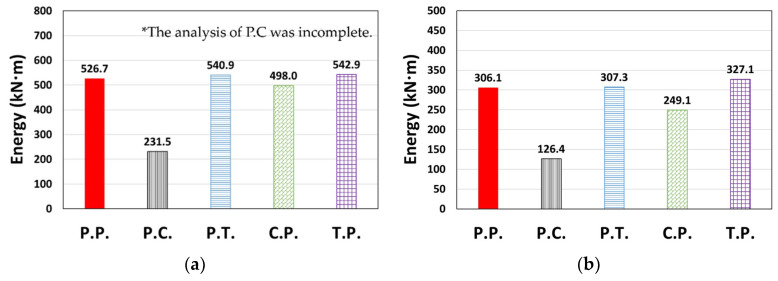

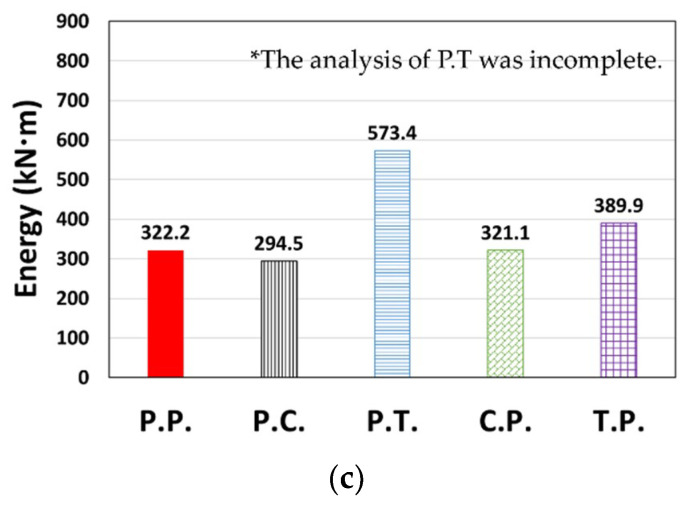
Total dissipated energy (*E_t_*): (**a**) Type 3 (Northridge, X-dir.), (**b**) Type 3 (Northridge, Y-dir.), and (**c**) Type 2 (El Centro, X-dir.).

A case where the results for a ground motion (or direction) different from that shown in Figure 7 is presented in Figure 9. Contrary to the previous tendency, C.P. showed relatively small values when compared to P.P. in Figure 9b, whereas P.T. and T.P. showed larger values than P.P. in Figure 9c. *E_t_* is presented in Figure 10; the *E_t_* value of the P.T. model was up to 78.0% higher than that of P.P., and the T.P. model also showed higher results than P.P. (3.1–21.0%). In contrast, P.C. and C.P. showed up to 58.7% and 18.6% lower *E_t_* than P.P., respectively.

According to the NDA results, in terms of the entire frame, although the overall responses of the analysis model did not show significant differences from those of the reference model, P.P., the energy dissipation in some models showed a rather significant error. In terms of energy dissipation, the models that showed similar results to the reference model were in the orders of C.P., T.P., P.C., and P.T.

### 3.3. Column Members

**Figure 11:**
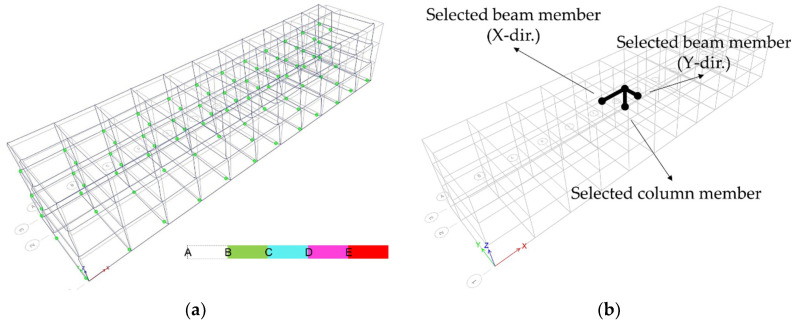
Type 1 (P.P, Northridge): (**a**) plastic hinge status and (**b**) selected structural members.

No distinct differences were found between the analysis models in the overall response. This section and the subsequent section present comparative analyses of the behavioral characteristics of the representative members constituting each model. Figure 11a illustrates an example of the plastic hinge distribution for Type 1, in which members beyond the yield strength, which is point B in the legend, are indicated. The analysis models, in which many plastic hinges were formed on the frame member, were examined to distinctly identify the differences in member behaviors depending on the applied hysteresis model to select a representative member. The members with large deformations were selected in various analysis models, as shown in Figure 11b.

**Figure 12:**
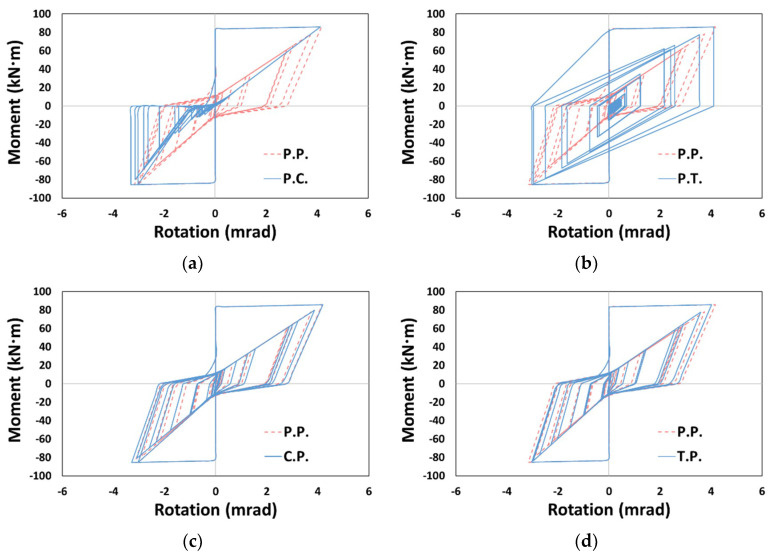
Moment–rotation curve (Type 1, Northridge, X-dir.): (**a**) P.C., (**b**) P.T., (**c**) C.P., and (**d**) T.P.

As shown in Figure 12, the moment–rotation curves in the column member exhibited a distinct difference from P.P. in P.C. and P.T. models, in which the pivot model was not applied to the column. Excessive pinching was observed in P.C. depending on the application of the concrete model, and relatively small pinching was observed in P.T. depending on the application of the Takeda model, similar to the results of a prior study [25].

**Figure 13:**
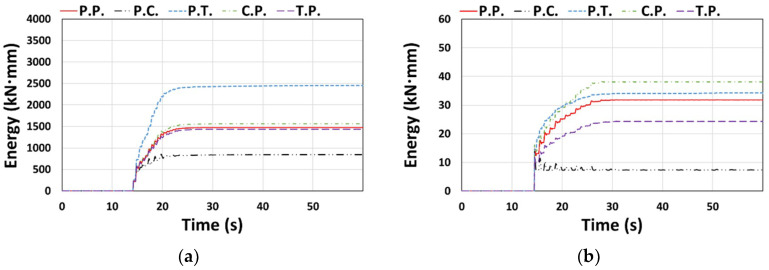

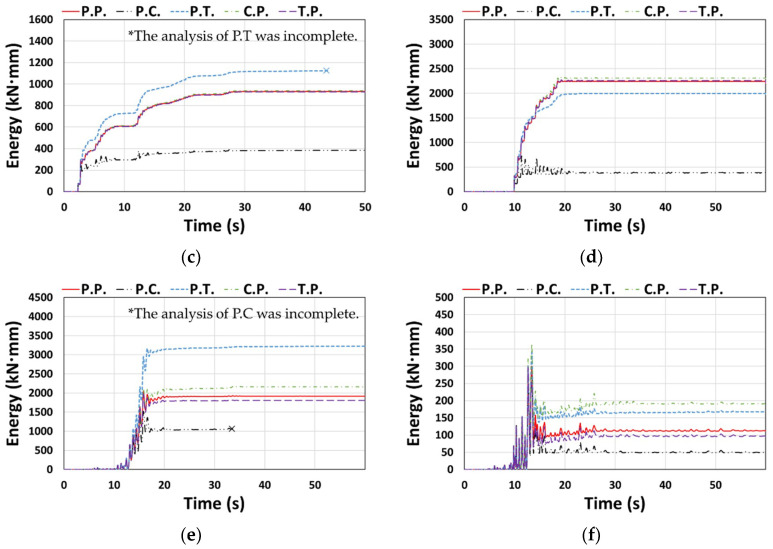
Cumulative dissipated energy: (**a**) Type 1 (Northridge, X-dir.), (**b**) Type 1 (Northridge, Y-dir.), (**c**) Type 2 (El Centro, X-dir.), (**d**) Type 2 (Northridge, Y-dir.), (**e**) Type 3 (Northridge, X-dir.), and (**f**) Type 3 (Northridge, Y-dir.).

**Figure 14:**
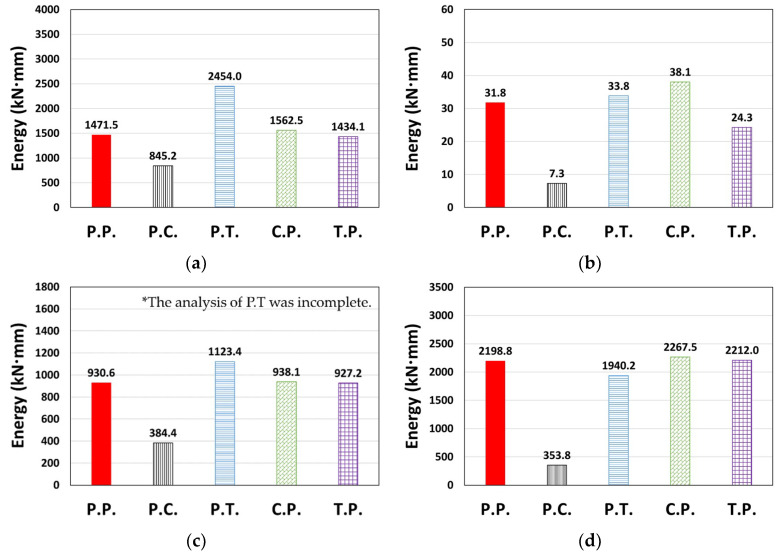

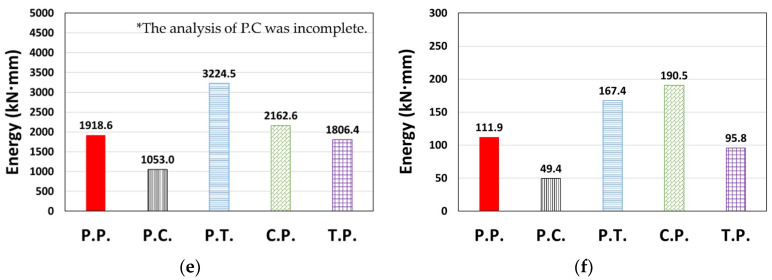
Total dissipated energy (*E_t_*): (**a**) Type 1 (Northridge, X-dir.), (**b**) Type 1 (Northridge, Y-dir.), (**c**) Type 2 (El Centro, X-dir.), (**d**) Type 2 (Northridge, Y-dir.), (**e**) Type 3 (Northridge, X-dir.), and (**f**) Type 3 (Northridge, Y-dir.).

For the entire structure shown in Section 3.2, P.C. and C.P. dissipated less energy, and P.T. and T.P. dissipated more energy than P.P., depending on the degree of pinching implemented in the concrete and Takeda models (Figure 9 and Figure 10). In contrast, C.P. tended to dissipate more energy and T.P. tended to dissipate less energy than P.P. for the column member (Figure 13), and P.T. dissipated considerably lower energy than P.P., as shown in Figure 13d. *E_t_* is shown in Figure 14. The *E_t_* values of C.P. and P.T. were up to 70.2% and 68.1% higher than that of P.P., respectively, whereas P.C. and T.P. showed up to 83.9% and 23.6% lower *E_t_*, respectively. For the column members, the models in the orders of T.P., C.P., P.T., and P.C. were compared as models with similar moment–rotation curves with the reference model P.P. having small differences in *E_t_*.

The energy dissipation of column members, depending on the applied hysteresis model, showed slightly different patterns when compared to the results of a previous study [25] and those of the entire frame; this was presumably because the beams and columns were connected and behaved in relation to each other within the frame. For example, P.C. showed low energy dissipation in the column, owing to the influence of the concrete model applied to the column, and the energy was mainly dissipated by the beam. Therefore, the *E_t_* of the column in P.C. was lower than that in P.P. Likewise, the relative difference in energy dissipation characteristics, evaluated using each hysteresis model applied to the beam and column members, influenced the energy dissipation of individual frame members. Moreover, it can be expected that the energy dissipation characteristics of the beam members are opposite to those of the column members.

### 3.4. Beam Members

**Figure 15:**
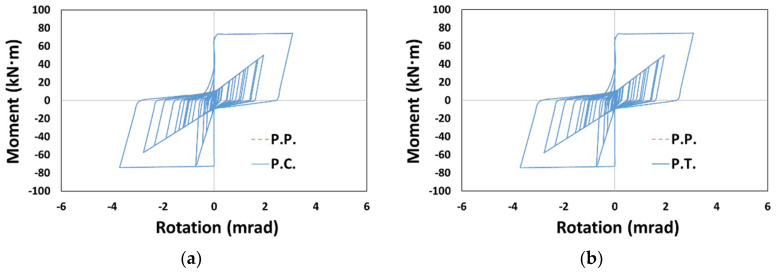

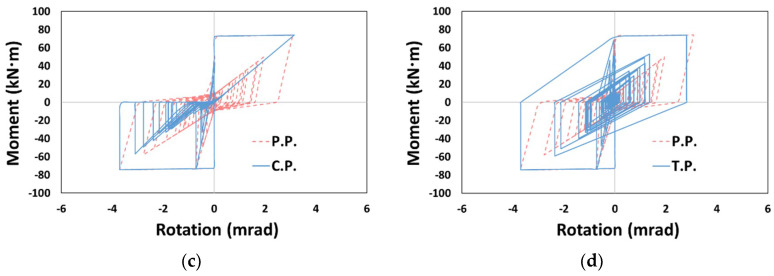
Moment–rotation curve (Type 1, Northridge, Y-dir.): (**a**) P.C, (**b**) P.T., (**c**) C.P., and (**d**) T.P.

**Figure 16:**
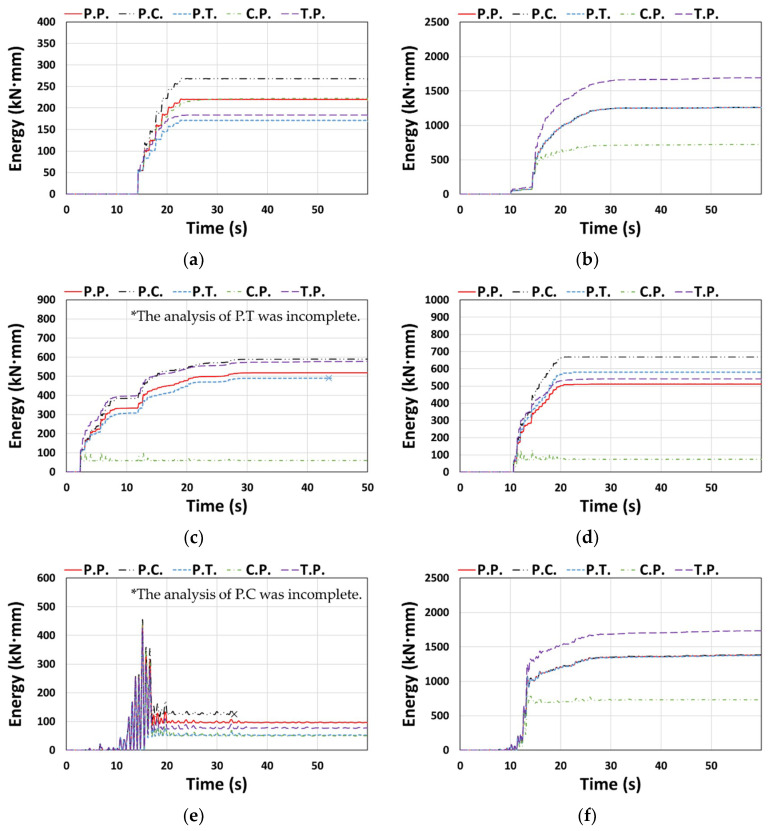
Cumulative dissipated energy: (**a**) Type 1 (Northridge, X-dir.), (**b**) Type 1 (Northridge, Y-dir.), (**c**) Type 2 (El Centro, X-dir.), (**d**) Type 2 (Northridge, Y-dir.), (**e**) Type 3 (Northridge, X-dir.), and (**f**) Type 3 (Northridge, Y-dir.).

The moment–rotation curves for the beam member are as shown in Figure 15, in which C.P. and T.P., without applying the pivot model to the beam, showed significant differences to the reference model P.P. As for the cumulative dissipation energy, Figure 16 shows that C.P. and P.T. generally had smaller values than those of P.P., whereas P.C. and T.P. showed larger values (there were exceptional cases where the value of T.P. was lower than that of P.P. (Figure 16a,e) or the value of P.T. was larger than that of P.P. (Figure 16d)).

**Figure 17:**
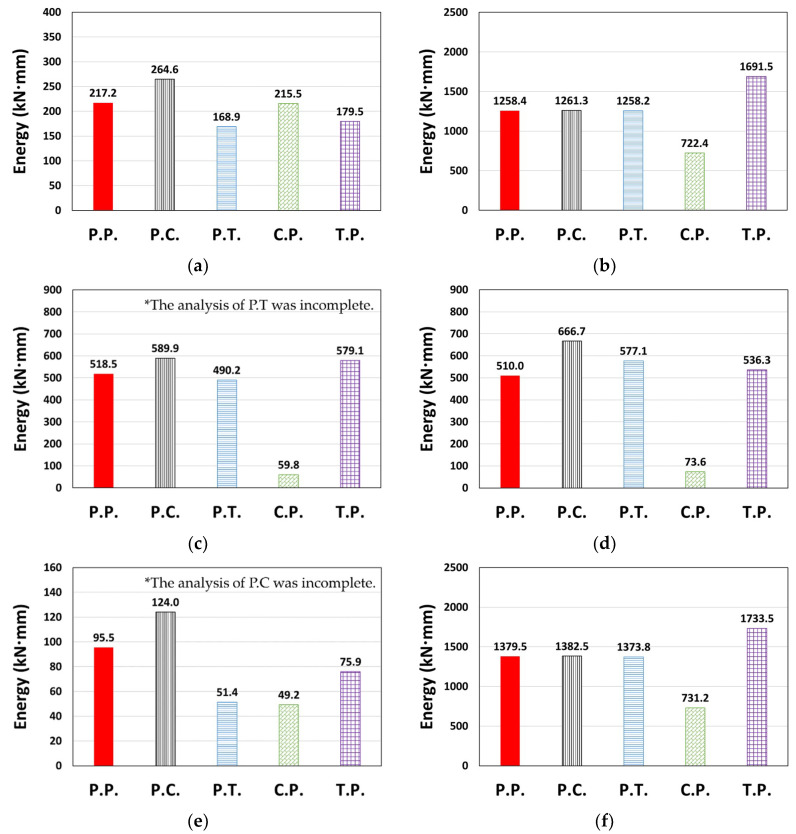
Total dissipated energy (*E_t_*): (**a**) Type 1 (Northridge, X-dir.), (**b**) Type 1 (Northridge, Y-dir.), (**c**) Type 2 (El Centro, X-dir.), (**d**) Type 2 (Northridge, Y-dir.), (**e**) Type 3 (Northridge, X-dir.), and (**f**) Type 3 (Northridge, Y-dir.).

Figure 17 illustrates the *E_t_* values; the *E_t_* of the P.C. model was up to 30.7% higher than that of P.P. T.P. also exhibited a maximum difference of 34.4%, showing a higher result than P.P. In contrast, C.P. and P.T. showed up to 88.5% and 46.2% lower *E_t_* values than P.P., respectively. In terms of energy dissipation, the models with small differences to the P.P. model were in the orders of P.C., T.P., P.T., and C.P., and the energy dissipation characteristics of the beam members were generally opposite to those of the column members, as predicted earlier.

### 3.5. Evaluation of an Appropriate Hysteresis Model

Regarding the behavior under quasi-static cyclic loading, a previous study reported that the pivot model showed results that were close to the experimental results [25]. Based on the comparison of the behavior characteristics of individual members using NDA in this study, the T.P. model was the one that evaluated the energy dissipation most similarly (showing a similar moment–rotation curve) to the reference model P.P. for the columns and P.C. for the beams. This result reconfirms that the most reliable hysteresis model for each structural member is the pivot model.

Although the pivot model can similarly predict the behavior of existing RC frame members in this way, parameters to represent the hysteresis characteristics need to be defined when applying them in an analysis model. Sharma et al. proposed parameters *α* related to the stiffness and *β* affecting pinching using regression analysis on a database of numerous seismic tests [21]. As shown in Equations (1) and (2), obtaining *α* and *β* requires calculating factors *k_a_* and *k_b_*, which are influenced by the reinforcement detailing of the sections and the applied axial load on the members. In other words, considerable time and effort are required to develop an analysis model because each member must be input separately after calculating the longitudinal reinforcement ratio, volumetric shear reinforcement ratio, and axial load ratio of all the members. The practicality of applying the pivot model can be significantly limited if the number of members increases as the scale of the analysis structure increases. Therefore, a hysteresis model that does not require parameter setting or is simple to set parameters while showing relatively reliable results is also required from a practical perspective.

(1) $$\alpha =0.170k_{a}+0.415$$

(2) $$\beta =0.485k_{b}+0.115$$

In this study, C.P. and T.P. were the analysis models that showed results that were close to the reference model P.P. in terms of the response of the entire frame (Section 3.2). In the two models, the analysis performed when applying C.P. and T.P. shortened the time required for identifying the characteristics of the structures and analysis computations compared to P.P., as the concrete and Takeda hysteresis models, applied to the beams, do not require parameter settings or are relatively simple for applying parameters, respectively [20]. In contrast, T.P. (Section 3.3) and P.C. (Section 3.4) were evaluated as the most suitable models for predicting column and beam behaviors, respectively. This result and those for the frame comprehensibly prove that the hysteresis model applied to the column has a greater influence on the overall behavior than in the case of the beam. Based on these comparisons and considerations, the T.P. model, which has the narrowest variation range and achieved the best prediction of the average response compared to the P.P. model in the evaluation of energy dissipation, is considered an applicable practical alternative in situations where it is difficult to apply the pivot model to all the frame members.

## 4. Conclusions

A study was conducted to identify an appropriate hysteresis model for NDA of existing RC moment frames. The pivot, concrete, and Takeda models were applied differently to beams and columns for the three types of structures analyzed, and NDA was performed in the X and Y directions for 45 analysis models involving the three ground motions. The results were comparatively analyzed in terms of the entire frame and the individual members, and the conclusions are summarized as follows:

(1) All analysis results showed the most significant differences in terms of energy dissipation, and the order in which the response of the entire frame was close to that of the reference model P.P. was in the orders of C.P., T.P., P.T., and P.C. In addition, T.P. for columns and P.C. for beams were found to be the analysis models that evaluated the energy dissipation similarly while having similar moment–rotation curves to those of P.P. Accordingly, the pivot model was considered the most appropriate hysteresis model for each structural member.

(2) Although the energy dissipation of the entire frame was considerably influenced by the dissipation characteristics of the concrete and Takeda models themselves, the energy dissipation of individual members was related to the relative difference in the dissipation characteristics evaluated by each hysteresis model applied to beams and columns. Likewise, the energy dissipation pattern of individual members, depending on the applied hysteresis model, is different from that of the entire frame as the connected beams and columns behave in relation to each other within the frame.

(3) Although the pivot model was the most reliable hysteresis model for both beams and columns as observed in preceding studies, parameters related to reinforcement detailing and applied loads need to be predefined to apply them to the analysis model. The T.P. model, which best predicted the average response while showing a narrow variation in the energy dissipation evaluation compared to P.P., could be a practical alternative in situations where it is difficult to apply the pivot model to all frame members.

## Figures and Tables

**Table 1:** Overview of analysis structures.

Classification	Type 1	Type 2	Type 3
Material strength	*f*^′^*_c_* = 21 MPa, *f_y_* = 300 MPa		
Story height (m)	3.3		
Story	3	1	3

Note: *f′_c_* and *f_y_* denote the compressive strength of concrete and yield stress of reinforcing bars, respectively.

**Table 2:** Description of the analysis models considered by type.

Analysis Model	Applied Hysteresis Model		Remarks
	Beam	Column	
P.P.	Pivot	Pivot	Reference model
P.C.	Pivot	Concrete	Column hysteresis model changed
P.T.	Pivot	Takeda	
C.P.	Concrete	Pivot	Beam hysteresis model changed
T.P.	Takeda	Pivot	

**Table 3:** Comparison of NSA and NDA results.

Response	NSA	NDA		
		El Centro	Northridge	Taft
Base shear (kN)	2327.4	2792.5	2639.5	1196.0
Roof displacement (mm)	80.7	87.3	86.7	46.6

**Table 4:** Summary of overall responses (*V_max_* and *D_max_*).

Model			*V_max_* (kN)		*D_max_* (mm)		Remarks
			X-dir.	Y-dir.	X-dir.	Y-dir.	
Type 1 (1000 y return period)	El Centro	P.P.	2792.5	2419.7	87.3	47.0	Elastic behavior in Y direction
		P.C.	2807.5	2419.7	90.3	47.0	
		P.T.	2780.4	2419.7	83.5	47.0	
		C.P.	2806.9	2419.7	88.5	47.0	
		T.P.	2774.5	2419.7	86.2	47.0	
	Northridge	P.P.	2639.5	2879.0	86.7	63.9	-
		P.C.	2638.8	2879.0	86.4	63.9	
		P.T.	2637.2	2879.0	85.7	63.9	
		C.P.	2643.7	2876.7	86.8	63.9	
		T.P.	2634.2	2875.2	85.8	63.8	
	Taft	P.P.	1196.0	1546.6	46.6	33.0	Elastic behavior in both X and Y directions
		P.C.	1196.0	1546.6	46.6	33.0	
		P.T.	1196.0	1546.6	46.6	33.0	
		C.P.	1196.0	1546.6	46.6	33.0	
		T.P.	1196.0	1546.6	46.6	33.0	
Type 2 (2400 y return period)	El Centro	P.P.	1996.6	1587.6	70.5	46.3	Analysis of X direction in P.T. was incomplete
		P.C.	2001.6	1710.5	70.7	46.3	
		P.T.	1975.3	1532.5	70.7	46.3	
		C.P.	1997.5	1589.4	70.5	46.3	
		T.P.	1996.4	1588.1	70.4	46.3	
	Northridge	P.P.	2008.2	3751.1	55.7	62.0	Elastic behavior in X direction
		P.C.	2008.2	3751.1	55.7	64.7	
		P.T.	2008.2	3751.1	55.7	62.6	
		C.P.	2008.2	3751.1	55.7	62.3	
		T.P.	2008.2	3751.2	55.7	61.9	
	Taft	P.P.	1162.1	1186.8	43.3	36.5	Elastic behavior in both X and Y directions
		P.C.	1162.1	1186.8	43.3	36.5	
		P.T.	1162.1	1186.8	43.3	36.5	
		C.P.	1162.1	1186.8	43.3	36.5	
		T.P.	1162.1	1186.8	43.3	36.5	
Type 3 (1000 y return period)	El Centro	P.P.	1768.4	2727.0	72.6	104.4	Elastic behavior in X direction
		P.C.	1768.4	2737.9	72.6	104.8	
		P.T.	1768.4	2723.8	72.6	103.2	
		C.P.	1768.4	3041.6	72.6	118.0	
		T.P.	1768.4	2424.2	72.6	98.4	
	Northridge	P.P.	2757.5	2470.6	124.2	82.4	Analysis of X direction in P.C. was incomplete
		P.C.	2753.7	2475.8	125.5	82.6	
		P.T.	2757.0	2471.6	120.7	82.0	
		C.P.	2764.7	2604.1	125.3	86.7	
		T.P.	2744.0	2387.5	122.4	76.1	
	Taft	P.P.	1155.0	1349.3	46.1	48.9	Elastic behavior in both X and Y directions
		P.C.	1155.0	1349.3	46.1	48.9	
		P.T.	1155.0	1349.3	46.1	48.9	
		C.P.	1155.0	1349.3	46.1	48.9	
		T.P.	1155.0	1349.3	46.1	48.9	

## Data Availability

The data presented in this study are available on request from the corresponding author. The data are not publicly available due to privacy.
